# Social Support and Its Influencing Factors Among Perimenopausal Women in Tianjin, China: A Community-Based Study

**DOI:** 10.3390/healthcare13091057

**Published:** 2025-05-04

**Authors:** Shuang Yuan, Jianping Ren

**Affiliations:** 1 School of Public Administration, Hangzhou Normal University, Hangzhou 311121, China; 2020011012001@stu.hznu.edu.cn; 2 Engineering Research Center of Mobile Health Management System, Ministry of Education, Hangzhou 311121, China

**Keywords:** perimenopausal women, social support, menopausal symptoms

## Abstract

Objectives: This study aimed to assess the social support level among perimenopausal women and explore its key influencing factors. Methods: From November 2022 to March 2023, a stratified multistage random sampling method was used to recruit 647 perimenopausal women from three communities in Tianjin, China. The participants completed the Social Support Rating Scale (SSRS), the Kupperman Menopausal Index (KMI), and a sociodemographic questionnaire. Nonparametric tests, correlation analysis, and stepwise regression analysis were conducted to explore key factors influencing social support. Robustness checks were performed using hierarchical regression analysis. Results: The overall social support level of perimenopausal women was moderately low (34.190 ± 10.007), with the lowest scores observed in the 46–50 age group (33.000 ± 9.666). Stepwise regression analysis showed that, compared to married women, single women reported significantly lower social support levels (β = −0.242, *p* < 0.001). Using public sector employees as the reference group, women in all other occupational categories (including self-employed, corporate employees, farmers, freelancers, and other professions) had significantly lower social support scores (β range: −0.196 to −0.232, all *p* < 0.05). Compared to those with good family relationships, women with average (β = −0.420, *p* < 0.001) and poor (β = −0.349, *p* < 0.001) family relationships reported significantly lower social support levels. In terms of menopausal symptoms, greater severity of palpitations (β = −0.140, *p* < 0.05) and dyspareunia (β = −0.143, *p* < 0.05) was associated with lower social support, while higher levels of neuroticism (β = 0.102, *p* < 0.05) and joint/muscle pain (β = 0.158, *p* < 0.05) were linked to greater social support. Conclusions: Social support levels among perimenopausal women were generally low, particularly among those aged 46–50 years. Marital status, occupational type, and family relationships were key influencing factors, and certain menopausal symptoms were closely related to social support, especially those that are difficult to discuss, such as palpitations and dyspareunia. These findings highlight the necessity of strengthening social support networks for perimenopausal women and provide scientific evidence for the development of targeted interventions and public health policies to enhance their well-being and promote healthy aging.

## 1. Introduction

As the global population continues to age, the health of perimenopausal women has emerged as a growing concern in public health research [1]. Perimenopause refers to the transitional phase from the initial decline in ovarian function and the onset of menstrual irregularities to one year after the final menstrual period [2]. Typically occurring between the ages of 40 and 55, this stage lasts approximately 4 to 10 years, depending on individual differences [3]. During perimenopause, women experience a marked decline in circulating estrogens, particularly estradiol (E2), accompanied by a compensatory increase in gonadotropins such as follicle-stimulating hormone (FSH) [4,5]. These hormonal fluctuations contribute to a broad spectrum of symptoms, including somatic complaints (e.g., hot flashes, sleep disturbances, palpitations, dyspareunia) and disturbances in psychosocial health (e.g., anxiety, depression, irritability, cognitive impairments) [4,6,7]. Currently, management of perimenopausal physiological and psychological symptoms primarily involves hormone replacement therapy (HRT), pharmacological treatments, such as antidepressants, lifestyle modifications, and psychosocial interventions [5,8,9]. Several studies have indicated that the perimenopausal period is a critical stage when psychophysiological distress reaches its peak. The burden resulting from a range of somatic symptoms during this phase may further contribute to the withdrawal of perimenopausal women from social activities, thereby weakening their social support networks [8,10]. Meanwhile, psychological symptoms during this stage not only impair women’s emotional resilience, but also diminish their ability to seek and maintain social support, thus exacerbating feelings of isolation and vulnerability [6,7,9].

In addition to symptom-related challenges, perimenopausal women often face significant psychosocial challenges, such as children becoming independent, aging parents passing away, and evolving familial responsibilities, all of which may further compromise emotional well-being and perceived social support [11]. Additionally, competing demands from work, family obligations, and societal expectations can deplete emotional resources [12]. Collectively, the interplay of biological, psychological, and social stressors positions perimenopause as a particularly vulnerable stage for women’s social well-being [8,9].

Social support, defined as the care, respect, and emotional and practical assistance perceived within one’s social networks, is a critical determinant of health, especially during periods of physiological transition and heightened stress [13]. It is typically categorized into three dimensions: subjective support (perceived emotional support and recognition), objective support (tangible or informational assistance), and support utilization (the extent to which individuals seek and use available support resources) [14]. Evidence suggests that these dimensions of social support are not only essential for well-being, but are also influenced by the individual’s health status [8,9].

According to the seminal framework by House, Umberson, and Landis, the relationship between social support and health is bidirectional: strong support improves health outcomes, while deteriorating health may lead to social withdrawal and reduced support, potentially forming a negative feedback loop [15]. In the context of perimenopause, social support has been shown to buffer the effects of stress and anxiety, mitigate symptom severity [16], and enhance self-efficacy, thereby improving overall quality of life [17]. Additionally, emotional regulation theory and stigma theory suggest that perimenopausal women may withdraw from social interactions or avoid seeking support due to anxiety, shame, or perceived stigma stemming from their symptoms [18].

Given the health and social implications of this life stage, understanding the status of social support among perimenopausal women and its influencing factors is both timely and necessary. Such understanding not only informs individualized care, but also contributes to public health strategies in the context of aging [1,5,8,9].

Although the importance of social support for menopausal women is well recognized, current research in China has largely treated menopause as a single, homogeneous phase [16,19]. Limited attention has been given to the distinct characteristics of perimenopause, particularly the nuanced relationship between specific symptoms and levels of social support [20]. This gap is especially pertinent in the Chinese sociocultural context, where traditional gender roles and familial obligations may influence women’s access to and use of support systems [21,22]. Addressing this gap, the present study aims to provide empirical evidence that can inform culturally tailored, targeted health interventions to promote well-being and healthy aging in this population.

Therefore, this study aimed to explore the level of social support among perimenopausal women and to identify its key influencing factors, with particular attention to the association between symptom severity and social support.

## 2. Methods

### 2.1. Participants and Procedure

This cross-sectional study was conducted as part of a community-based initiative aimed at improving health management strategies for perimenopausal women. Data collection took place between December 2022 and March 2023 in Tianjin, China. A multistage sampling strategy was utilized. Initially, three administrative districts were purposively selected based on demographic diversity and logistical feasibility. Subsequently, within each district, three subdistricts were chosen using purposive sampling to reflect a representative range of community characteristics.

Subsequently, a structured questionnaire survey was conducted among women aged 40 to 55 years living in the selected communities through convenience sampling. The questionnaire collected data on sociodemographic characteristics, menopausal symptoms—assessed using the Kupperman Menopausal Index (KMI)—and perceived social support—measured using the Social Support Rating Scale (SSRS).

The sample size was estimated using the calculation formula for a single sample mean. Based on a previous study, the social support score assessed with the same measure was 39.58 ± 7.31 [23] among community-dwelling perimenopausal women in Jinzhou City, Liaoning Province. A margin of error of 1 point was set. A nonresponse rate of 30% was also considered. We aimed to recruit at least 294 participants. Finally, 900 perimenopausal women were invited to participate, and 51 declined.

A total of 849 questionnaires were collected, of which 647 met the inclusion criteria for perimenopausal status and were included in the final analysis. The inclusion criteria were as follows: (1) women aged 40–55 years; (2) able to understand the questionnaire and provide informed consent; (3) self-reported changes in menstrual cycle length of more than 7 days on at least two occasions within the past 12 months; and (4) last menstrual period occurring within 12 months prior to the survey. The exclusion criteria included the following: (1) a history of severe physical or mental illness; (2) cognitive impairment; and (3) a history of surgical menopause or hysterectomy.

This study was approved by the Ethics Committee of the School of Public Health at Hangzhou Normal University (Approval No: 20220011). All participants provided written informed consent.

### 2.2. Sociodemographic Characteristics

This study used a self-developed questionnaire to collect demographic information, including age, educational level, marital status, number of children, occupation, personal monthly income, type of medical insurance, self-rated health status, and self-assessed relationship with family members. These variables were collected to understand the sociodemographic characteristics of the sample and their potential influence on this study’s findings.

### 2.3. Social Support Rating Scale (SSRS)

Social support among perimenopausal women was assessed using the Social Support Rating Scale (SSRS), developed by Xiao in 1986 [24]. The SSRS has been widely used in Chinese populations to evaluate social support in health-related research, and it has demonstrated good reliability and validity in studies involving menopausal women [25]. The scale consists of 10 items covering three dimensions: subjective support (4 items; range: 8–32), objective support (3 items; range: 1–22), and support utilization (3 items; range: 3–12). The total score ranges from 12 to 66, with higher scores indicating higher levels of perceived social support.

Subjective support refers to the individual’s emotional experience and satisfaction with being respected, understood, and supported. Objective support captures tangible or visible support, including material assistance, social networks, and group affiliations. Support utilization reflects the extent to which individuals participate in social activities and seek support when encountering adverse events.

In this study, the SSRS demonstrated good internal consistency, with a Cronbach’s alpha coefficient of 0.819.

### 2.4. Kupperman Menopausal Index (KMI)

Menopausal symptoms were assessed using the Kupperman Menopausal Index (KMI), a widely used instrument developed by Dr. Kupperman in 1953 [26]. The KMI has demonstrated high levels of reliability and validity in both clinical and research settings, with reported Cronbach’s alpha values typically exceeding 0.80 [27].

The scale consists of 11 common symptoms associated with menopause, including hot flashes, sweating, sleep disturbances, depression, irritability, dizziness, fatigue, joint and muscle pain, headaches, heart palpitations, and paresthesia (or formication). Each symptom is rated on a 4-point Likert scale ranging from 0 (no symptoms) to 3 (severe symptoms), and selected items are weighted according to their clinical significance—hot flashes, for instance, receive the highest weight. The total KMI score ranges from 0 to 51 and is commonly categorized into three levels of symptom severity: mild (0–14), moderate (15–24), and severe (≥25).

In this study, the KMI demonstrated excellent internal consistency, with a Cronbach’s alpha of 0.930.

### 2.5. Data Analysis

All study data were double-entered into an Excel database to ensure the accuracy of the data entered, with discrepancies resolved through comparison. The data were analyzed using IBM SPSS Statistics version 29.0 (IBM Corp., Armonk, NY, USA). Descriptive statistics were employed to summarize demographic characteristics and key variables, including means, standard deviations, frequencies, and percentages. The normality of data distribution was assessed using the Shapiro–Wilk test. Given that some variables did not follow a normal distribution, nonparametric tests were used to analyze differences in social support across various demographic groups.

Pearson correlation analysis was employed to explore the relationship between perimenopausal symptoms and social support. Stepwise linear regression (forward selection) was conducted to identify the most significant predictors of social support, while minimizing the risk of multicollinearity caused by the inclusion of too many independent variables.

Finally, hierarchical regression was employed as a sensitivity analysis to verify the robustness of the regression model, while simultaneously assessing the independent contribution of variables at each level to social support. In this model, demographic variables (e.g., age, marital status, and education level) were entered in the first layer as control variables, followed by self-rated health status in the second layer, self-assessed relationship with family members in the third layer, and perimenopausal symptom scores in the fourth layer. In all analyses, a *p*-value of less than 0.05 was considered statistically significant.

## 3. Results

### 3.1. Sociodemographic Characteristics of Participants

This study included a total of 647 participants with a mean age of 46.36 ± 3.54 years. The majority were married (68.8%) and had one child (68.0%). Most participants had an associate degree or higher (71.0%), and were primarily enterprise employees (29.8%) or self-employed (23.2%). Regarding personal monthly income, 66.5% of participants earned between CNY 3000 and 5999. The majority (78.8%) were covered by Urban Employee Basic Medical Insurance. In terms of self-rated health, 68.9% rated their health as “Good” or “Very Good,” and 60.6% reported “Good” family relationships. The mean Kupperman score was 18.31 ± 6.86, with 45.4% categorized as mild, 37.1% as moderate, and 17.5% as severe (Table 1).

### 3.2. Social Support Score

As shown in Table 2, the scores for each dimension of social support among perimenopausal women were as follows: the subjective support score was 18.65 ± 5.641, the objective support score was 6.09 ± 2.051, and the support utilization score was 6.87 ± 2.617. The total social support score was 34.19 ± 10.007. These scores reflect participants’ perceived social support across different dimensions.

### 3.3. Univariate Analysis of Demographic Characteristics and Social Support Dimensions

The univariate analysis of demographic characteristics and social support scores across various dimensions using nonparametric tests is summarized in Table 3. Significant associations were found between age and subjective support, support utilization, and total social support scores (*p* < 0.05), with women aged 51–55 reporting the highest subjective support scores. Married women had significantly higher subjective support, objective support, and total scores across all dimensions compared to those in the “Other” category (*p* < 0.001). Additionally, women with two or more children showed higher subjective support and total scores (*p* < 0.001).

Education level was significantly associated with all dimensions of social support (*p* < 0.001), where those with a bachelor’s degree or above had the highest total scores. Occupational status also influenced social support, with state employees reporting the highest scores across all dimensions (*p* < 0.001). While support utilization and objective support differed significantly across income levels (*p* = 0.013, *p* < 0.001), total social support scores were not significantly affected (*p* = 0.233).

Women with Urban Employee Basic Medical Insurance had higher scores across all dimensions compared to those with other or no insurance (*p* < 0.001). Self-rated health and family relationships were strongly associated with all social support dimensions (*p* < 0.001), with better health and stronger family relationships correlating with higher scores.

### 3.4. Correlation Between KMI Scores and Social Support Scores

To explore the relationship between the severity of perimenopausal symptoms and social support scores in various dimensions, Pearson correlation analysis was conducted, and the results were visualized using a correlation heatmap (Table 4). The analysis revealed that all perimenopausal symptoms were significantly correlated with the total social support score, subjective support, and objective support dimensions (*p* < 0.001), with all symptoms showing negative correlations except for joint and muscle pain. Nervousness and headache showed no significant correlation with the support utilization dimension (*p* > 0.05). Additionally, joint and muscle pain was positively correlated with all dimensions of social support.

### 3.5. Multifactor Analysis of Social Support Scores

Stepwise linear regression was performed using variables that showed significance in univariate analysis. Across the SSRS total score and its three dimensions (subjective support, objective support, and support utilization), age, marital status, occupation, self-rated family relationships, and perimenopausal symptoms were consistently identified as significantly associated factors, though their associations varied across dimensions.

First, in the multivariate regression analysis of overall social support scores (Table 5), it was found that women aged 46–50 years (β = −1.285, *p* = 0.037), those with non-marital statuses, such as unmarried, divorced, and widowed (β = −5.752, *p* < 0.001), and self-employed women (β = −3.565, *p* < 0.001), had lower scores. Additionally, poor family relationships had the strongest negative association (β = −11.356, *p* < 0.001). Education level had a relatively weaker influence, with women holding only associate degrees showing significantly lower scores (β = −1.547, *p* = 0.013). Among perimenopausal symptoms, palpitations (β = −2.593, *p* < 0.001) and dyspareunia (β = −1.283, *p* = 0.003) were associated with lower overall social support scores, while nervousness (β = 0.957, *p* = 0.025) and joint and muscle pain (β = 2.228, *p* < 0.001) were linked to higher scores.

In the subjective support analysis (Table 6), a similar pattern was observed. Age, marital status, and self-rated family relationships remained significant. Women aged 46–50 years (β = −0.680, *p* = 0.042) and those with non-marital statuses (β = −3.164, *p* < 0.001) reported lower subjective support scores. Notably, vertigo (β = 1.039, *p* = 0.004) was positively associated with subjective support, which differed from its effects in other dimensions. Education level was not significantly associated with subjective support in this dimension.

In the analysis of objective support (Table 7), while marital status and family relationships remained critical, urinary symptoms (β = 0.260, *p* = 0.047) and joint and muscle pain (β = 0.545, *p* < 0.001) showed positive associations, contrasting with their effects in other dimensions. Additionally, the occupational category “Others (e.g., workers, freelancers, β = −1.208, *p* < 0.001) had a more pronounced negative impact in this dimension compared to its influence on subjective support. Education level was not significantly associated with objective support in this dimension.

In the analysis of support utilization (Table 8), education level played a more significant role, with women holding a bachelor’s degree or higher reporting better utilization scores (β = 0.649, *p* = 0.003). Income levels also emerged as uniquely significant factors, with participants earning 6–9 K (β = 1.275, *p* < 0.001) and ≥9 K (β = 1.726, *p* < 0.001) reporting higher utilization scores. Nervousness (β = 0.552, *p* < 0.001) and depression (β = −0.588, *p* < 0.001) also played distinct roles in support utilization.

Overall, consistent patterns across all dimensions include the strong influence of marital status, occupation, and family relationships, while each dimension also revealed unique associations with specific symptoms and socioeconomic factors.

### 3.6. Sensitivity Analysis: Hierarchical Regression of Social Support Scores

To evaluate the stability of the stepwise regression model, hierarchical regression was conducted as a sensitivity analysis (Table 9). In Step 1, demographic variables explained 20.9% of the variance in social support (R^2^ = 0.209). Step 2 added self-rated health status, increasing the *R*^2^ to 26.9% (Δ*R*^2^ = 0.062, *p* < 0.01). Step 3 further improved the model by incorporating family relationship variables, which raised the R^2^ to 43.5% (ΔR^2^ = 0.163, *p* < 0.01). Finally, in Step 4, the inclusion of perimenopausal symptoms modestly increased the explanatory power to R^2^ = 0.461 (ΔR^2^ = 0.036, *p* < 0.001).

After controlling for all factors in Step 4, several variables remained significant. Compared to married women, unmarried women had significantly lower social support levels (β = −0.280, *p* < 0.01). For occupation, self-employed individuals, other employment types, and retirees reported significantly lower social support levels compared to state employees (*p* < 0.001). Monthly income was positively associated with social support, with individuals earning CNY 9000 or more reporting significantly higher levels than those earning CNY 2999 or less (β = 0.132, *p* < 0.001). Additionally, individuals with poorer family relationships had significantly lower social support levels compared to those with good family relationships (*p* < 0.01).

Among perimenopausal symptoms, dyspareunia (β = −0.143, *p* < 0.05) and palpitations (β = −0.140, *p* < 0.05) were negatively associated with social support, whereas joint and muscle pain (β = 0.158, *p* < 0.05) and nervousness (β = 0.102, *p* < 0.05) was positively associated. These findings indicate that the key predictors identified in the stepwise regression analysis remained significant and generally consistent in the hierarchical model, supporting the robustness of the regression model.

## 4. Discussion

This study examined the levels of social support and the severity of perimenopausal symptoms among 647 perimenopausal women living in Tianjin, China. The average age of participants was 46.36 ± 3.54 years, consistent with findings in related studies [28]. The total social support score was 34.19 ± 10.007, indicating a moderately low level relative to other age groups, as reported in similar research [16]. Analysis identified that age, marital status, occupation, family relationships, and the severity of specific perimenopausal symptoms were significantly associated with social support levels, which supported both Hypotheses 1 and 2.

Specifically, perimenopausal women aged 46–50 years reported lower levels of social support. This reduction may be associated with a contraction in their social networks, possibly influenced by shifts in social roles—such as children becoming independent and parents aging or passing away—which leads to a loss of regular emotional support sources [29]. Women in this age group are often seen as typical of the ‘sandwich generation’, bearing dual responsibilities of caring for aging parents while supporting partially independent children, placing them in a ‘sandwiched’ position between generations [30]. This dual role imposes significant psychological stress, limiting their time and capacity to seek and maintain social support. In the Chinese context, this phenomenon is particularly pronounced. Societal expectations to ‘care for parents’ and ‘support children’, alongside traditional gender roles assigning primary family responsibilities to women, further intensify the psychological burden on perimenopausal women, reducing their opportunities to access needed support [31]. Additionally, this age group represents the peak period for perimenopausal symptoms. Research shows that perimenopausal women aged 46–50 are more likely to experience symptoms such as hot flashes, irritability, dyspareunia, and joint and muscle pain [16,32]. These symptoms are associated with challenges in women’s psychological state and social interactions, which may make it more difficult to seek and receive social support.

Marital status and family relationships are also significantly associated with social support levels, consistent with previous findings [16]. Married women typically receive more emotional support and practical help from spouses, family members, and shared social circles. In contrast, unmarried, divorced, or widowed women may face a reduction in social support sources [16]. Furthermore, good family relationships usually mean higher emotional support and interaction, which is particularly important during the perimenopausal stage [16,33]. We also found that self-employed individuals, retirees, and other occupational groups, including freelancers, reported significantly lower levels of social support compared to government officials and company employees. This discrepancy may stem from less stable income sources, fewer social security services, and the nature of their work, which limits access to stable social settings, making it difficult to establish long-term social relationships, resulting in less support [34,35]. Especially after retirement, social support networks shrink rapidly, leading to a significant decline in social support levels [36]. Additionally, higher educational attainment and personal income were associated with greater support utilization. Women with higher education typically possess better cognitive skills and access to information, making them more proficient at seeking and utilizing social support resources when faced with challenges. Research indicates that highly educated women maintain a more positive attitude towards perimenopause and are more effective at seeking support for menopausal symptoms [37]. Similarly, women with higher incomes usually have more resources and opportunities to expand their social networks, allowing them to flexibly utilize social support systems when experiencing perimenopausal symptoms [38].

This study also observed notable patterns regarding the association between the severity of perimenopausal symptoms and levels of social support. Specifically, women experiencing more severe dyspareunia and palpitations reported lower social support levels. This may be because palpitations typically manifest as sudden rapid heartbeats and chest tightness, which can occur during social activities, leading women to develop fear or avoidance of social situations [39]. Additionally, symptoms like palpitations may be misunderstood by family members as excessive nervousness or anxiety, resulting in emotional communication barriers. In such cases, women may feel they have insufficient understanding and support from their families, further reducing their perceived social support [40]. Dyspareunia not only affects women’s physical health, but also directly impacts intimate relationships. This discomfort may lead women to avoid sexual activities, which can make partners feel rejected, resulting in emotional distancing and communication barriers, thereby reducing the perceived care and support within intimate relationships [16]. Research shows that sexual disharmony is a significant cause of tension in intimate relationships, and when intimate relationships are strained due to dyspareunia, the support and care women feel at home also decrease [41]. This is an important aspect of understanding the social support issues faced by perimenopausal women. In the context of Chinese culture, due to a generally conservative attitude toward sexual issues [42], perimenopausal women may be reluctant to express concerns related to sexual health, such as dyspareunia. Because of embarrassment or concerns about social judgment, women are less likely to seek help in family or medical settings, further limiting their access to social support.

Conversely, this study found that the severity of some symptoms, such as nervousness and joint/muscle pain, was positively correlated with social support levels. This may be because these symptoms are more common and easily recognizable, making women more willing to seek help and more likely to receive support from family, friends, or medical resources. This proactive help-seeking behavior increases their opportunities for interaction with others, thereby allowing them to gain more social support [43]. This is consistent with findings from related studies that certain perimenopausal symptoms can elicit a stronger demand for support [38]. This could explain why paresthesia and depressive symptoms were negatively correlated with objective support levels and support utilization, respectively. Paresthesia, being difficult to describe, may cause women to be reluctant or unwilling to seek help, leading to reduced objective support [44]. Depressive symptoms can cause women to feel helpless, lack motivation, and even be skeptical of support systems, making it difficult for them to effectively utilize available social support resources [44]. Research shows that depression not only reduces perceived social support, but also weakens the ability to actually use these supports. Even when social support is available, women in a depressive state may not actively seek help, leading to a significant reduction in support utilization [45]. Notably, this study found that vertigo was positively correlated with subjective support, suggesting that subjective support may be more dependent on women’s subjective perceptions of their conditions and their ability to express their needs, rather than directly depending on the severity of the symptoms [37].

The stepwise regression analysis in this study identified marital status, occupation, family relationships, and the severity of certain perimenopausal symptoms as key factors significantly associated with social support levels. However, R^2^ values across models ranged from 0.212 to 0.472, indicating that these variables account for only a portion of the variance in social support. To further validate the model’s robustness, hierarchical regression was conducted as a sensitivity analysis, yielding results largely consistent with the stepwise regression. Key factors, including marital status, occupation, family relationships, and specific symptoms (e.g., nervousness, dyspareunia, joint and muscle pain, and palpitations), remained significant after controlling for additional variables, confirming model stability. Although the final model’s R^2^ was 0.461, indicating limited explanatory power, the study reliably elucidates significant associations with social support, providing a robust foundation for understanding the social support needs of perimenopausal women.

## 5. Conclusions

This study found that social support levels among perimenopausal women in China were generally low, with the lowest levels observed among those aged 46–50. Social support was significantly associated with marital status, occupation, family relationships, and specific perimenopausal symptoms, particularly those that are less apparent or more difficult to articulate, such as palpitations and dyspareunia. These findings underscore the urgent need to strengthen social support networks for perimenopausal women, especially in the face of culturally specific barriers to emotional expression and help-seeking. Importantly, this study provides scientific evidence to inform the development of targeted interventions and public health policies aimed at enhancing well-being and promoting healthy aging in this vulnerable population.

### 5.1. Intervention Recommendations

To optimize intervention strategies, community and familial support for perimenopausal women should be strengthened. Community initiatives could include perimenopausal support groups, mental health services, and family relationship counseling programs, which can help women manage physiological and psychological challenges. Such expanded social support systems are particularly beneficial for unmarried women and those with weaker family relationships. Additionally, workplace interventions like flexible working hours and health benefits can create a more supportive environment, facilitating easier access to social support.

### 5.2. Clinical and Cultural Sensitivity

Clinical interventions should prioritize symptoms such as palpitations and dyspareunia, which are often challenging to articulate. Given that expressions related to ‘sexual health’ are more reserved within East Asian cultural contexts, and since sexual quality of life is closely linked to social support levels, there is a pressing need for increased clinical sensitivity to these symptoms. Such measures could facilitate more targeted medical support, improve quality of life, and promote the mental well-being of perimenopausal women.

### 5.3. Policy Implications

Within the Chinese cultural context, traditional gender roles and societal expectations add caregiving responsibilities for perimenopausal women, especially those in the ‘sandwich generation’. These pressures intensify psychological burdens and limit access to social support. To address this, public education and awareness initiatives by government and health agencies are essential to reduce misconceptions and foster a more supportive environment. Integrating support systems for perimenopausal women into community healthcare and workplace health policies can further alleviate the social and psychological burdens on this group.

### 5.4. Future Research

Future research should explore barriers to social support among perimenopausal women in diverse cultural and geographical contexts, including urban and rural settings. Such studies should consider the role of communities and healthcare providers in supporting this population. Additionally, longitudinal research is necessary to identify key factors influencing perimenopausal experiences and long-term health outcomes. These studies, along with subsequent interventions, are crucial for optimizing health management during this transitional stage, and should be integrated into comprehensive public health strategies that promote healthy aging across the life course.

## 6. Limitations

While this study revealed key factors influencing social support among perimenopausal women, several limitations should be acknowledged. First, as this is a cross-sectional study, it cannot establish causal relationships between social support and perimenopausal symptoms. Future research should employ longitudinal designs to explore the dynamic impact of social support on perimenopausal symptoms. Second, the sample was limited to three administrative districts in Tianjin, China, and may not represent perimenopausal women from other regions of China, particularly those in rural areas. Future studies should expand the sample to enhance the generalizability and applicability of the findings across different regions and cultural contexts. The measurement of social support primarily relied on self-report scales, which may introduce subjective bias, such as over- or underestimation of perceived social support. Future studies should combine objective indicators and multi-dimensional assessment methods to improve the reliability of the measurements. Although this study conducted a sensitivity analysis using hierarchical regression to verify the robustness of the regression model and accounted for some potential confounders, such as demographic variables and perimenopausal symptoms, other variables, such as mental health status and broader social networks, may not have been fully controlled. Future studies should address these factors by incorporating more independent variables to further strengthen the robustness and credibility of the findings.

## Figures and Tables

**Table 1:** General socioeconomic and health characteristics of the study population (*N* = 647).

Characteristic	*n*	%
Age (years), mean ± SD	46.36 ± 3.535	
40–45	241	37.2
46–50	342	52.9
51–55	64	9.9
Marital status		
Married	445	68.8
Other	202	31.2
Number of children		
0	101	15.6
1	440	68.0
2+	106	16.4
Education		
Junior High School or Below	65	10.0
High School	123	19.0
Associate Degree	232	35.9
Bachelor’s Degree or Above	227	35.1
Occupation		
State Employee	119	18.4
Enterprise Employee	193	29.8
Self-Employed	150	23.2
Other	104	16.1
Retired Personnel	81	12.5
Personal Monthly Income (CNY)		
0–2999	25	3.9
3000–5999	430	66.5
6000–8999	145	22.4
≥9000	47	7.3
Type of medical insurance		
Urban Employee Basic Medical Insurance	510	78.8
Other	137	21.2
Self-Rated Health Status		
Very good	252	38.9
Good	194	30.0
Fair	161	24.9
Poor or Very Poor	40	6.2
Self-Rated Family Relationship		
Good	392	60.6
Fair	177	27.4
Poor	78	12.1
Kupperman score (mean ± SD)	18.31 ± 6.86	
Mild (0–14)	294	45.4
Moderate (15–24)	240	37.1
Severe (≥25)	113	17.5

Note: In the marital status category, “Other” includes never married, divorced, and widowed individuals. In the occupation category, “Other” includes workers, farmers, fishermen, and freelancers. In the type of medical insurance category, “Other” includes individuals with other types of insurance, such as New Rural Cooperative Medical Insurance (NRCMI), Urban Resident Basic Medical Insurance (URBMI), commercial insurance, and those without any insurance. The ranges of personal monthly income are inclusive, meaning that both the lower and upper bounds are included in each category (e.g., CNY 3000–5999 includes incomes of CNY 3000 and CNY 5999). The currency unit is Chinese Yuan (CNY); approximate equivalents in U.S. dollars (USD) are based on an exchange rate of CNY 1 ≈ USD 0.14 at the time of data collection.

**Table 2:** Social support total score and dimensions (mean ± SD).

Social Support Dimensions	Scores (Mean ± SD)
Subjective support	18.65 ± 5.641
Objective support	6.09 ± 2.051
Support utilization	6.87 ± 2.617
Total scores	34.19 ± 10.007

**Table 3:** Social support scores across different sociodemographic and health-related groups (*N* = 647).

Characteristic	Subjective Support M (P_25_, P_75_)	t/H/Z	Objective Support M (P_25_, P_75_)	t/H/Z	Support Utilization M (P_25_, P_75_)	t/H/Z	Total Score M (P_25_, P_75_)	t/H/Z
Age (years)		12.962 *		0.291		10.616		10.469
40–45	18.000 (15.0, 24.0)		6.000 (5.0, 7.0)		7.000 (5.0, 9.0)		35.000 (28.0, 43.0)	
46–50	17.000 (14.0, 22.0)		6.000 (5.0, 7.0)		6.000 (5.0, 8.0)		31.000 (27.0, 40.0)	
51–55	20.000 (15.0, 24.0)		6.000 (5.0, 7.0)		6.000 (5.3, 8.0)		34.000 (29.0, 42.0)	
Marital status		−9.204 **		−9.428 **		−2.277 *		−8.842 **
Married	19.000 (15.0, 25.0)		6.000 (5.0, 8.0)		7.000 (5.5, 9.0)		35.000 (29.0, 44.0)	
Other	15.000 (12.0, 18.0)		5.000 (3.0, 6.0)		6.000 (4.0, 8.0)		28.000 (22.0, 34.3)	
Number of children		17.030 **		26.954 **		4.099		12.594 *
0	16.000 (13.0, 20.0)		5.000 (3.5, 6.0)		7.000 (5.0, 9.0)		30.000 (24.0, 38.0)	
1	17.500 (14.0, 24.0)		6.000 (5.0, 7.0)		7.000 (5.0, 8.0)		32.000 (27.0, 42.0)	
2+	19.000 (15.8, 25.0)		6.000 (5.0, 7.3)		7.000 (4.8, 10.0)		35.000 (29.0, 44.3)	
Education		28.908 **		15.640 **		24.137 **		30.210 **
Junior High School or Below	15.000 (12.0, 19.0)		5.000 (5.0, 6.0)		6.000 (4.0, 9.0)		29.000 (23.0, 31.5)	
High School	18.000 (15.0, 23.0)		6.000 (5.0, 8.0)		6.000 (6.0, 8.0)		33.000 (28.0, 42.0)	
Associate Degree	17.000 (14.0, 23.0)		6.000 (5.0, 7.0)		7.000 (4.0, 8.0)		31.000 (27.0, 40.0)	
Bachelor’s Degree or Above	20.000 (15.0, 25.0)		6.000 (5.0, 7.0)		7.000 (6.0, 10.0)		36.000 (28.0, 44.0)	
Occupation		27.276 **		43.526 **		39.660 **		41.135 **
State Employee	19.000 (15.0, 25.0)		6.000 (5.0, 8.0)		8.000 (6.0, 10.0)		37.000 (29.0, 45.0)	
Enterprise Employee	19.000 (15.0, 24.0)		6.000 (5.0, 8.0)		7.000 (6.0, 9.0)		35.000 (28.5, 44.5)	
Self-Employed	16.000 (13.0, 19.0)		5.000 (5.0, 7.0)		6.000 (5.0, 8.0)		30.000 (25.0, 34.0)	
Others	17.000 (14.5, 23.0)		5.000 (5.0, 7.0)		6.000 (4.0, 7.0)		31.000 (26.0, 40.5)	
Retired Personnel	18.000 (14.0, 24.0)		5.000 (5.0, 6.0)		6.000 (4.0, 8.0)		30.000 (26.0, 42.0)	
Personal Monthly Income (CNY)		3.791		10.855 *		30.790 **		4.274
0–2999	17.000 (12.5, 21.0)		5.000 (5.0, 6.0)		6.000 (3.0, 7.0)		30.000 (23.5, 34.0)	
3000–5999	18.000 (14.0, 24.0)		6.000 (5.0, 8.0)		6.000 (5.0, 8.0)		32.000 (27.0, 43.0)	
6000–8999	17.000 (14.0, 21.0)		6.000 (5.0, 7.0)		7.000 (5.0, 10.0)		33.000 (28.0, 40.0)	
≥9000	18.000 (15.0, 23.0)		6.000 (5.0, 6.0)		8.000 (6.0, 9.0)		31.000 (28.0, 42.0)	
Type of medical insurance		−4.979 **		−4.972 **		−3.605 **		−5.706 **
Urban Employee Basic Medical Insurance	18.000 (15.0, 24.0)		6.000 (5.0, 8.0)		7.000 (6.0, 9.0)		34.000 (28.0, 43.0)	
Other	16.000 (13.0, 19.0)		5.000 (5.0, 6.5)		6.000 (3.5, 8.0)		29.000 (24.0, 34.0)	
Self-Rated Health Status		85.728 **		27.206 **		26.997 **		73.583 **
Very good	20.000 (16.0, 25.0)		6.000 (5.0, 9.0)		7.000 (6.0, 9.0)		37.000 (29.0, 47.0)	
Good	18.000 (15.0, 23.3)		6.000 (5.0, 7.0)		7.000 (5.0, 9.0)		32.000 (28.8, 42.0)	
Fair	15.000 (13.0, 18.0)		6.000 (5.0, 7.0)		6.000 (4.0, 8.0)		29.000 (24.5, 34.0)	
Poor or Very Poor	14.000 (11.0, 18.5)		5.000 (3.3, 6.0)		5.500 (3.0, 7.8)		27.000 (21.0, 32.5)	
Self-Rated Family Relationship		249.766 **		121.108 **		36.856 **		212.415 **
Good	22.000 (17.0, 25.8)		6.000 (5.0, 8.0)		7.000 (6.0, 9.0)		39.000 (31.0, 46.0)	
Fair	15.000 (13.0, 17.0)		5.000 (5.0, 6.0)		6.000 (4.0, 8.0)		29.000 (24.0, 31.0)	
Poor	12.500 (10.0, 16.0)		5.000 (2.0, 5.0)		6.000 (3.0, 8.0)		24.500 (18.0, 29.3)	

Note: Main variable values are reported to three decimal places for consistency, while values within parentheses (P_25_, P_75_) are reported with minimal necessary decimal places to enhance table clarity. Nonparametric tests were used for the analysis: Kruskal–Wallis H test for comparisons among multiple groups (H value) and Mann–Whitney U test for comparisons between two groups (Z value). For a detailed explanation of the “Other” categories, please refer to the note in Table 1. * *p* < 0.05; ** *p* < 0.01.

**Table 4:** Correlation analysis between perimenopausal symptoms (KMI) scores and social support scores.

Perimenopausal Symptoms	Subjective Support	Objective Support	Support Utilization	Total Score
Hot Flashes and Sweating	−0.137 **	−0.122 **	−0.075	−0.142 **
Paresthesia	−0.223 **	−0.164 **	−0.124 **	−0.221 **
Insomnia	−0.355 **	−0.254 **	−0.175 **	−0.341 **
Nervousness	−0.265 **	−0.281 **	−0.063	−0.269 **
Urinary Symptoms	−0.313 **	−0.226 **	−0.139 **	−0.304 **
Dyspareunia	−0.306 **	−0.260 **	−0.159 **	−0.315 **
Depression	−0.325 **	−0.248 **	−0.177 **	−0.322 **
Vertigo	−0.107 **	−0.132 **	−0.061	−0.120 **
Fatigue	−0.175 **	−0.147 **	−0.098 *	−0.180 **
Joint and Muscle Pain	−0.208 **	−0.151 **	−0.097 *	−0.204 **
Headache	−0.140 **	−0.145 **	−0.067	−0.148 **
Palpitations	−0.245 **	−0.180 **	−0.145 **	−0.248 **
Formication	−0.209 **	−0.218 **	−0.089 *	−0.224 **
Total Score	−0.324 **	−0.272 **	−0.155 **	−0.327 **

* *p* < 0.05, ** *p* < 0.001.

**Table 5:** Multifactor analysis of social support scores (total score).

Variable	β	t	*p*	95% CI
Constant	44.408	51.429	<0.001	42.715~46.100
Age (ref = 40–45 years)				
46–50	−1.285	−2.094	0.037	42.715~46.100
Marital Status (ref = Married)				
Other	−5.752	−8.467	<0.001	−2.488~−0.082
Occupation (ref = State Employee)				
Self-Employed	−3.565	−4.671	<0.001	−2.761~−0.332
Others	−4.968	−5.637	<0.001	−5.060~−2.069
Retired Personnel	−3.813	−4.046	<0.001	−6.695~−3.240
Education (ref = Junior High School or Below)				
Associate degree	−1.547	−2.496	0.013	−7.084~−4.421
Self-Rated Family Relationship (ref = good)				
Fair	−9.780	−13.776	<0.001	−5.660~−1.966
Poor	−11.356	−10.263	<0.001	−11.172~8.389
Perimenopausal Symptoms				
Palpitations	−2.593	−4.551	<0.001	−3.710~−1.477
Nervousness	0.957	2.246	0.025	−13.524~9.187
Joint and Muscle Pain	2.228	3.551	<0.001	−2.114~−0.452
Dyspareunia	−1.283	−3.025	0.003	0.122~1.792
*R* ^2^	0.445			

**Table 6:** Multifactor analysis of social support scores (subjective support).

Variable	β	t	*p*	95% CI
Constant	23.147	48.287	<0.001	22.208~24.087
Age (ref = 40–45 years)				
46–50	−0.680	−2.033	0.042	−1.336~−0.024
Marital Status (ref = Married)				
Other	−3.164	−8.479	<0.001	−3.896~−2.433
Occupation (ref = State Employee)				
Self-Employed	−0.870	−2.155	0.032	−1.661~−0.079
Self-Rated Family Relationship (ref = Good)				
Fair	−5.899	−15.523	<0.001	−6.644~−5.154
Poor	−6.817	−12.649	<0.001	−7.873~−5.761
Perimenopausal Symptoms				
Palpitations	−1.244	−4.455	<0.001	−1.791~−0.697
Vertigo	1.039	2.884	0.004	0.333~1.745
*R* ^2^	0.472			

**Table 7:** Multifactor analysis of social support scores (objectives).

Variable	β	t	*p*	95% CI
Constant	7.760	42.268	<0.001	7.400~8.120
Marital Status (ref = Married)				
Other	−1.339	−8.891	<0.001	1.634~1.043
Occupation (ref = State Employee)				
Self-Employed	−0.645	−3.818	<0.001	0.977~0.314
Others	−1.208	−6.338	<0.001	1.582~0.835
Retired Personnel	−0.565	−2.694	0.007	0.976~0.154
Self-Rated Family Relationship (ref = Good)				
Fair	−1.381	−8.608	<0.001	1.696~1.067
Poor	−1.839	−7.731	<0.001	2.306~1.373
Perimenopausal Symptoms				
Urinary Symptoms	0.260	1.987	0.047	0.003~0.517
Dyspareunia	−0.401	−3.164	0.002	0.650~0.153
Joint and Muscle Pain	0.545	3.938	<0.001	0.274~0.816
Palpitations	−0.291	−2.215	0.027	0.548~0.033
Formication	−0.287	−2.389	0.017	0.522~0.052
*R* ^2^	0.370			

**Table 8:** Multifactor analysis of social support scores (support utilization).

Variable	β	t	*p*	95% CI
Constant	7.660	28.500	<0.001	7.133~8.187
Age (ref = 40–45 years)				
46–50	−0.470	−2.430	0.015	0.850~0.091
Marital Status (ref = Married)				
Other	−0.427	−1.972	0.049	0.852~0.003
Education (ref = Junior High School or Below)				
Bachelor’s Degree or Above	0.649	3.008	0.003	0.852~0.003
Income (ref = 0–2999 CNY)				
6000–8999	1.275	5.264	<0.001	0.800~1.750
≥9000	1.726	4.418	<0.001	0.960~2.492
Occupation (ref = State Employee)				
Self-Employed	−1.099	−4.133	<0.001	1.620~0.578
Others	−1.718	−6.042	<0.001	2.275~1.161
Retired Personnel	−1.129	−3.679	<0.001	1.730~0.527
Self-Rated Family Relationship (ref = Good)				
Fair	−1.001	−4.419	<0.001	1.445~0.557
Poor	−1.248	−3.600	<0.001	1.928~0.569
Perimenopausal Symptoms				
Nervousness	0.552	4.126	<0.001	0.290~0.815
Depression	−0.588	−3.509	<0.001	0.917~0.260
*R* ^2^	0.212			

**Table 9:** Hierarchical regression analysis of factors influencing social support scores.

		Dependent Variable: Total Social Support Score							
		Model 1		Model 2		Model 3		Model 4	
		SE	β	SE	β	SE	β	SE	β
Step 1	Constant	3.074	-	2.990	-	2.643	-	2.818	-
	Age (ref = 40–45 years)								
	46–50	0.800	−0.105 *	0.784	−0.053	0.689	−0.067	0.690	−0.062
	51–55	1.425	0.032	1.380	0.061	1.218	0.022	1.218	0.001
	Marital Status (ref = Married)								
	Other	0.861	−0.299 **	0.841	−0.280 **	0.748	−0.231 **	0.755	−0.242 **
	Number of children (ref = 0)								
	1	1.120	0.083	1.084	0.070	0.953	0.061	0.940	0.052
	2+	1.344	0.122 *	1.302	0.095 *	1.148	0.053	1.136	0.070
	Education (ref= Junior High School or Below)								
	High School	1.421	0.159 *	1.379	0.109 *	1.223	0.026	1.226	0.044
	Associate degree	1.318	0.100	1.273	0.081	1.137	−0.030	1.143	−0.007
	Bachelor’s Degree or Above	1.452	0.229 *	1.400	0.201 *	1.255	0.050	1.253	0.069
	Occupation(ref = State Employee)								
	Enterprise Employee	1.194	0.023	1.154	−0.010	1.015	−0.019	0.996	−0.017
	Self-Employed	1.482	−0.105	1.427	−0.111	1.262	−0.188 **	1.244	−0.196 **
	Others	1.356	−0.137 *	1.305	−0.127 *	1.159	−0.197 **	1.165	−0.232 **
	Retired Personnel	1.523	−0.125 *	1.482	−0.140 *	1.306	−0.116 *	1.292	−0.126 *
	Income (ref = 0–2999 CNY)								
	3000–5999	1.945	0.040	1.889	0.065	1.662	0.053	1.643	0.073
	6000–8999	2.075	0.098	2.019	0.059	1.777	0.059	1.748	0.083
	≥9000	2.320	0.149 *	2.249	0.117 *	1.977	0.126 *	1.957	0.132 *
	Type of medical insurance (ref = Other)								
	Urban Employee Basic Medical Insurance	1.247	−0.106 *	1.206	−0.073	1.075	0.025	1.075	0.022
Step 2	Self-Rated Health Status (ref = Very Good)								
	Good			0.846	−0.132 *	0.764	−0.028	0.808	−0.017
	Fair			0.912	−0.277 **	0.896	−0.053	1.161	−0.035
	Poor or Very Poor			1.535	−0.154 **	1.405	−0.048	2.065	−0.039
Step 3	Self-Rated Family Relationship (ref = Good)								
	Fair					0.797	−0.420 **	0.820	−0.420 **
	Poor					1.062	−0.357 **	1.173	−0.349 **
Step 4	Perimenopausal Symptoms								
	Hot Flashes and Sweating							0.941	−0.003
	Paresthesia							0.822	0.018
	Insomnia							0.617	−0.067
	Nervousness							0.469	0.102 *
	Urinary Symptoms							0.634	0.076
	Dyspareunia							0.587	−0.143 *
	Depression							0.705	−0.064
	Vertigo							0.884	0.045
	Fatigue							0.778	0.092
	Joint and Muscle Pain							0.755	0.158 *
	Headache							0.834	−0.047
	Palpitations							0.654	−0.140 *
	Formication							0.567	−0.049
	*R* ^2^	0.209		0.269		0.435		0.461	
	*F*	11.655 **		13.510 **		24.675 **		17.246 **	
	Δ*R*^2^	0.228		0.062		0.163		0.036	
	Δ*F*	11.655 **		18.290 **		93.048 **		3.321 **	

* *p* < 0.05, ** *p* < 0.001.

## Data Availability

The datasets generated and/or analyzed during the current study are not publicly available due to the ethical and privacy restrictions of questionnaire surveys, but are available from the corresponding author on reasonable request.

## Abbreviations

The following abbreviations are used in this manuscript:

SSRS	Social Support Rating Scale
KMI	Kupperman Menopausal Index
